# Plasma dilution improves cognition and attenuates neuroinflammation in old mice

**DOI:** 10.1007/s11357-020-00297-8

**Published:** 2020-11-15

**Authors:** Melod Mehdipour, Taha Mehdipour, Colin M. Skinner, Nathan Wong, Chao Liu, Chia-Chien Chen, Ok Hee Jeon, Yi Zuo, Michael J. Conboy, Irina M. Conboy

**Affiliations:** 1 Department of Bioengineering and QB3, UC Berkeley, Berkeley, CA USA; 2 Department of Molecular and Cellular Biology and QB3, UCSC, Santa Cruz, CA USA; 3 Buck Institute for Research on Aging, 8001 Redwood Boulevard, Novato, CA USA; 4 Department of Biomedical Sciences, Korea University College of Medicine, Seoul, Republic of Korea

**Keywords:** Plasma dilution, Neuroinflammation, Neutral blood exchange, Senolytics, Memory and Cognition, Rejuvenation

## Abstract

Our recent study has established that young blood factors are not causal, nor necessary, for the systemic rejuvenation of mammalian tissues. Instead, a procedure referred to as neutral blood exchange (NBE) that resets signaling milieu to a pro-regenerative state through dilution of old plasma, enhanced the health and repair of the muscle and liver, and promoted better hippocampal neurogenesis in 2-year-old mice (Mehdipour et al., *Aging* 12:8790–8819, 2020). Here we expand the rejuvenative phenotypes of NBE, focusing on the brain. Namely, our results demonstrate that old mice perform much better in novel object and novel texture (whisker discrimination) tests after a single NBE, which is accompanied by reduced neuroinflammation (less-activated CD68^+^ microglia). Evidence against attenuation/dilution of peripheral senescence-associated secretory phenotype (SASP) as the main mechanism behind NBE was that the senolytic ABT 263 had limited effects on neuroinflammation and did not enhance hippocampal neurogenesis in the old mice. Interestingly, peripherally acting ABT 263 and NBE both diminished SA-βGal signal in the old brain, demonstrating that peripheral senescence propagates to the brain, but NBE was more robustly rejuvenative than ABT 263, suggesting that rejuvenation was not simply by reducing senescence. Explaining the mechanism of the positive effects of NBE on the brain, our comparative proteomics analysis demonstrated that dilution of old blood plasma yields an increase in the determinants of brain maintenance and repair in mice and in people. These findings confirm the paradigm of rejuvenation through dilution of age-elevated systemic factors and extrapolate it to brain health and function.

**Supplementary Information:**

The online version contains supplementary material available at 10.1007/s11357-020-00297-8.

## Introduction

Heterochronic parabiosis is a procedure where a young animal and an old animal are surgically conjoined so that the two partners share a common circulatory system after a certain amount of time [1–3]. Parabiosis studies have yielded a plethora of insights regarding mechanisms that underlie the aging of stem cell niches. It was shown that old partners have better health in multiple tissues when they shared blood with a younger animal [1, 4, 5]. A prominent interpretation of heterochronic parabiosis is that aging is malleable and that the aging process can be slowed or even reversed [1, 6]. With this, heterochronic parabiosis studies seeded the field of systemic aging and rejuvenation.

Brain aging in particular is associated with a progressive loss of functionality and is thought to be in large part the result of an excessive activation of microglia, the brain-resident myeloid cells [7]. The age-related declines in brain function and cognition (among many other functions in the body) were once considered inevitable and permanent [8]. Parabiosis studies, interestingly, have challenged this notion by illustrating the plasticity of brain maintenance and function after changing the age of the blood and also providing the environmental enrichment and shared young organs to the old partners [5, 9, 10].

Several systemic proteins and young plasma infusions were suggested to influence the plasticity of brain aging [9, 11–17], albeit with some controversy to the actual age-specific levels of some of these candidate factors, such as GDF11, B2M, CCL11, and TIMP2 [9, 18–24]. There was also a lack of health span increase in young plasma infusion studies [25]; and while safety trials were successful, the young blood approaches have not been demonstrated to be effective in improving the health of the brain or any other tissue in clinic [26]. In concert, heterochronic blood transfusion exchange experiments have shown that in the absence of the organ sharing and environmental enrichment of parabiosis, young blood does not rejuvenate the old brain [21].

As we investigate and form an evolutionary conserved paradigm of systemic rejuvenation, our data demonstrated that young blood is not the primary determinant, and instead, dilution of old blood plasma yields a robust resetting of the systemic signaling milieu to youth and health, rejuvenating multiple tissues [18]. The study of the brain in that report was limited to hippocampal neurogenesis; here we expand the work to other important facets of brain health: neuroinflammation and cognition. Our data demonstrate that neuroinflammation (specifically the activation of microglia), declines and the cognitive capacity of old mice (novel object and novel texture tests), improves after a single NBE. In correlation with the phenotypes of rejuvenated brain health and function, our comparative proteomics analysis revealed specific neuroprotective, neurogenic, and neuroactivity regulating proteins that become systemically upregulated in mice and people after plasma dilution. Considering that therapeutic plasma exchange (TPE) is FDA approved, this study suggests a use of this procedure to prevent, attenuate, and possibly even reverse the degenerative and inflammatory diseases of the brain.

## Results

### Cognitive capacity of old mice is improved through a single NBE procedure

**Fig. 1:**
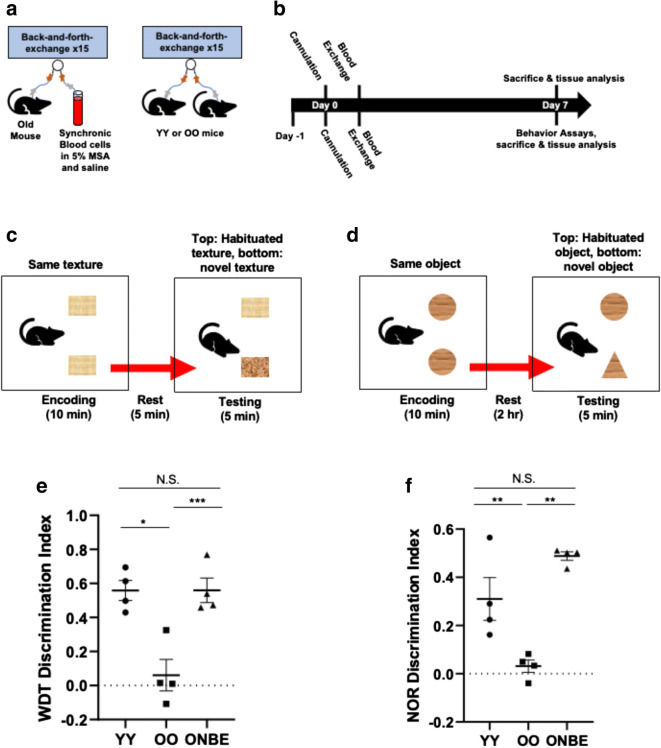
NBE quickly and robustly improves the cognitive performance of old mice. **a** Experimental schematic. Catheters were installed into the jugular veins of young or old mice, which had 50% of their blood plasma exchanged with normal saline (0.9% sodium chloride), 5% mouse serum albumin (MSA), and synchronic blood cells, as previously published [21]. **b** Timeline. At day 1, mice were habituated for whisker discrimination (WD) and novel object recognition (NOR) behavioral tests. These mice underwent jugular vein cannulation on day 0 and blood exchange at day 1. WD and NOR assays were performed on day 6. Blood samples and brain were collected for tissue analysis. **c** Schematics of mice performing the whisker discrimination task and **d** novel object recognition assays. **e** OO mice performed poorly when compared to YY mice, as expected (*p value = 0.024). ONBE mice were much better at discriminating between habituated and novel textures versus OO animals (***p value < 0.00001). Interestingly, ONBE mice were able to discriminate between novel and habituated textures as effectively as YY mice (N.S. *p* value = 0.01). **f** Similar trends were observed with novel object recognition studies. YY versus ONBE N.S. *p* value = 0.99, YY versus OO **p value < 0.004, OO versus ONBE **p value < 0.006. *p* values were obtained by one-tailed Student’s *t* test, e.g., as typical for evaluating the extent of the differences in the means from three independent treatment groups, rather than comparing each group to another. *N* of YY = 4, *N* of OO = 4, and *N* of ONBE = 4 for each behavioral assay

Young (2–4 months) and old (22–24 months) male C57/B6 mice underwent one NBE procedure, as published [18]. Isochronic exchanges between young mice (young exchanged with young, YY) and old mice (old exchanged with old, OO) were performed, as controls for the procedure. Six days after the single NBE, or control YY or OO blood exchange, brain neuroinflammation assays, cognitive performance tests, and blood proteomics assays were performed, as in [21, 27–29] (Fig. 1a, b).

Cognitive changes occur naturally during the process of mammalian aging, typically associating with a decline in learning and memory [30]. Aged mammals exhibit poor cognitive capacity to detect and interact with novel textures and novel objects, as compared to much better inquisitiveness in such settings by young mammals [9, 10, 15, 29, 31, 32]. This aspect of cognition, which also reflects the quality of short-term memory, can be examined in the whisker discrimination (WD) task that tests sensory processing through the barrel cortex and memory through the hippocampus [32, 33] and by administering the novel object recognition (NOR) test [28], where sensory and memory information is processed through both the hippocampus and the perirhinal regions [34]. Aged mice typically have deficiency in short-term memory that compounds their inability to distinguish between different textures and different objects [29].

Whisker discrimination (Fig. 1c) and novel object recognition (Fig. 1d) tests were performed after an NBE to old mice, as in [29], using OO isochronically exchanged mice as controls for the procedure and YY isochronically exchanged mice, as standards for healthy young cognitive capacity. As expected, OO mice had a profound age-specific decline in their NOR and WD test performance, as compared to YY animals (Fig. 1e, f). However, a single dilution of old plasma resulted in much improved cognitive performance of old mice, which became, in fact, similar to that of the YY cohort (Fig. 1e and f). The encoding phase for each of these behavioral studies is shown in Supplementary Figure 1.

These results establish that functionality of the old brain (short memory and inquisitiveness) is robustly and rapidly improved by the dilution of old blood plasma.

### Age-associated increase in neuroinflammation is significantly attenuated by NBE

Neuroinflammation increases with age in mice and humans, which contributes to the decline in cognitive capacity of old mammals [9, 15, 29]. Thus, we studied if cognitive improvements in the old mice after NBE correlated with the diminished neuroinflammation.

To this end, we performed immunofluorescence on CD68, the marker of activated microglia, in 25-μm serial brain cryosections of young and old mice at 6 days post NBE or OO isochronic exchange, as well as the positive control for low neuroinflammation, YY exchanged mice.

**Fig. 2:**
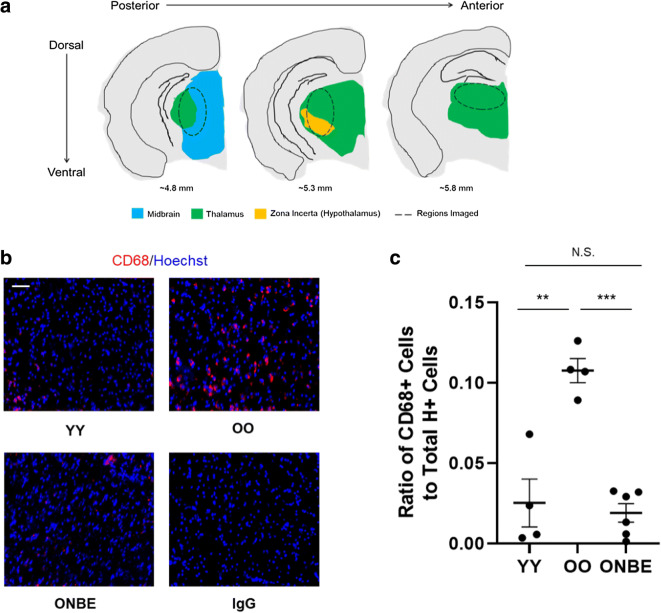
Neuroinflammation is reduced by a single NBE in old mice. **a** Schematic of serial sections of 25 μm (denoted by the dashed ovals) that were taken from regions ventral to the dentate gyrus, e.g., where an apparent age-associated increase in relative number of CD68^+^ cells was detected (green: thalamus, blue: midbrain, and orange: zona incerta of the thalamus). **b** Immunofluorescence was performed to assay for CD68-positive (red) activated microglia in the thalamus/hypothalamus/midbrain regions of brains from mice of each cohort. Representative CD68/Hoechst double-positive cells in the specified areas are shown for YY, OO (isochronic controls), and ONBE mice. Isotype-matched IgG negative controls show the absence of non-specific fluorescence. Scale bar 50 μm. **c** Quantification of the relative frequency of CD68^+^/Hoechst^+^ activated microglia in the thalamus. Neuroinflammation is substantially reduced in ONBE mice when compared to that in OO mice (***p value < 0.00002). The relative numbers of activated microglia are not significantly different between YY mice and ONBE mice (N.S. *p* value = 0.27). **p value YY versus OO < 0.003. *p* values were obtained by two-tailed Student’s *t* test. *N* of YY = 4, *N* of OO = 4, *N* of ONBE = 7

Figure 2a illustrates an anatomical map depicting various structures in coronal mouse brain sections (adapted from the Allen Brain Atlas) that were examined for the presence of CD68^+^ cells. Sections were collected from the plane of maximally exposed dentate gyrus (DG) of the hippocampus. Activated CD68^+^ (high) microglial cells were found just beneath (ventral) to the DG part of the brain, in the thalamus, midbrain, and zona incerta of the thalamus in the OO brains. These areas of the sections were imaged; and other parts of the OO brains were profiled, as well, but did not have detectable CD68^+^ microglial cells. YY and ONBE brain sections were identically studied, e.g., throughout the brain in serial 25-μm cryosections.

As shown in Fig. 2b and quantified in C, CD68^+^ cells were numerous in the old control brains (OO) and minimal/non-existent in young control brains (YY), which is consistent with the previously published age increase of CD68^+^ brain-resident cells [10, 17, 29]. Importantly, neuroinflammation became significantly diminished in old mice after one NBE. The representative and quantified images are of the areas that are depicted in Fig. 2a; profiling the entire YY and ONBE brains by immunofluorescence microscopy did not reveal CD68^+^ cells at other locations, either. The non-specific immunofluorescence of isotype-matched IgG controls was negligible (Fig. 2b).

These results demonstrate that neuroinflammation becomes significantly and quickly diminished in the brains of old mice after a single large volume dilution of blood plasma by NBE.

### ABT 263 senolytic and NBE both reduce brain senescence, but ABT 263 fails to enhance neurogenesis or robustly reduce neuroinflammation in the old mice

To examine whether and to what degree the positive effects of NBE might be emulated by ablation of senescent cells, we performed studies with ABT 263.

ABT 263 (Navitoclax) is a chemotherapy drug that is used to induce apoptosis in cervical, esophageal, leukemia, and lung cancer cells by inhibiting the anti-apoptotic proteins, Bcl-2 and Bcl-xL [35–38]. ABT 263 was shown to selectively clear senescent cells in vivo through inhibition of Bcl proteins [39–41] and to diminish SASP [42].

ABT drugs have been tested on patient glioblastomas in culture [43]; but these molecules are generally considered to be too large to cross the blood–brain barrier (BBB) [44]. Yet, the BBB becomes more porous with age even to such large proteins as albumin [45–47]. Additionally, peripheral inflammation has been reported to influence central inflammation and brain health [48–50]. Peripheral systemic senescence-associated secretory phenotype (SASP) proteins traverse the aged BBB and cause neuroinflammation and immune infiltration [45, 46, 51, 52].

We therefore hypothesized that peripheral senescent cell clearance by ABT 263 might diminish the levels of SASP systemically, preventing their mobilization to the brain, thereby improving hippocampal neurogenesis and attenuating neuroinflammation in old mice. And we were interested to determine whether a dilution of systemic SASP by NBE might attenuate the load of senescent cells in the old brain.

**Fig. 3:**
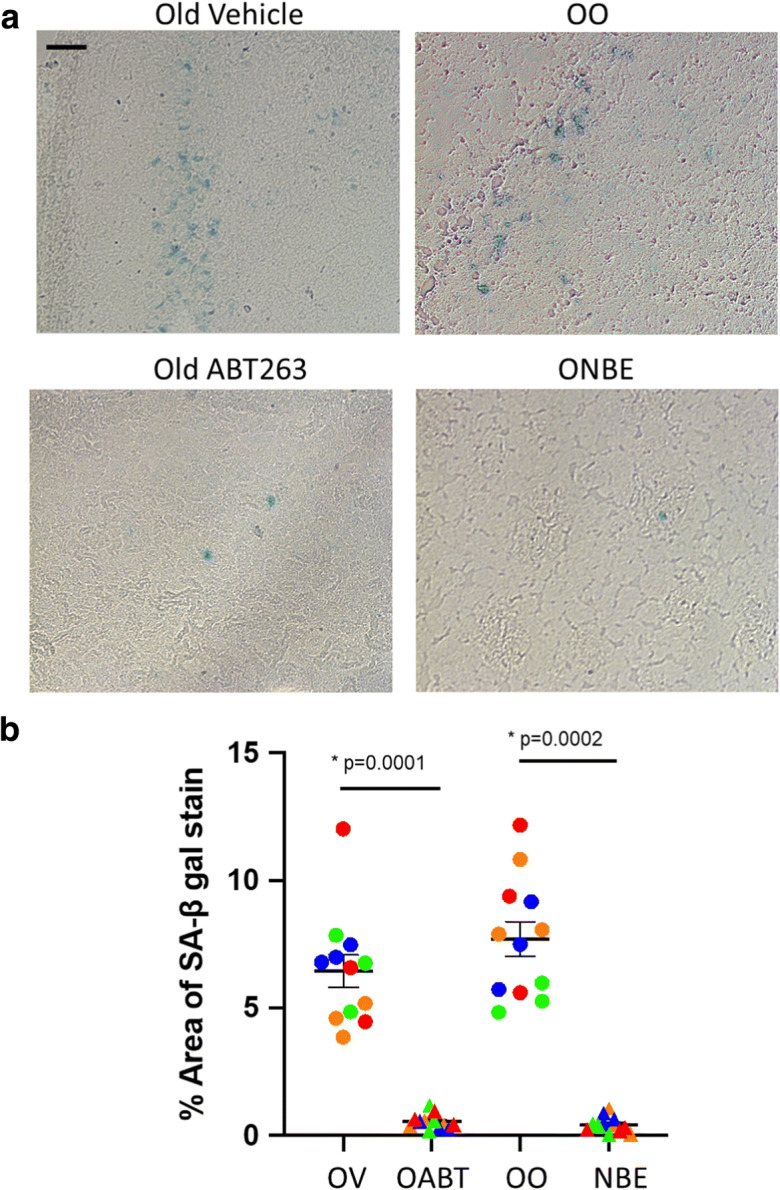
ABT 263 and NBE both reduce brain senescence. Freshly frozen 25-μm cryosections of old brains were assayed by SA-βGal, as per manufacturer’s instructions (9860S, Cell Signaling Technology). **a** Representative images of SA-β-gal staining, scale bar = 50 μm. **b** Percent SA-β-gal-positive areas per section were quantified and compared between the cohorts. Senescence of the old brain was diminished by both ABT 263 (ABT) and NBE, as compared to the vehicle control (OV) and isochronic old-to-old blood exchanges (OO). *p* values were obtained by two-tailed Student’s *t* test. ABT 263 versus vehicle *p* = 0.0001, NBE versus OO *p* = 0.0002. *N* = 4 animals of each cohort. Color coding illustrates the distribution of SA-βGal-positive cells in serial brain sections of each examined animal where the same color represents serial sections of the same brain (circles, vehicle or OO; triangles, ABT 263 or NBE)

To test these hypotheses, we compared the effects of ABT 263 and NBE, using the SA-βGal assay on brain sections. ABT 263 or vehicle control was administered to old C57/B6 mice (22–24 months of age) by gavage for two 7-day cycles with a 2-week time interval between each cycle at 50 mg per kg of body weight per day. NBE and control isochronic OO exchanges were performed, as described above. Freshly frozen 25-μm brain sections were assayed by senescence-associated β-galactosidase (SA-β-gal) staining, which revealed that both ABT 263 and NBE significantly reduced the load of senescent cells in the old brains, as compared to the vehicle control for ABT 263 and OO control exchanges for NBE (Fig. 3a, b).

Thus, interestingly, peripherally acting senolytic and dilution of old systemic milieu both reduce brain senescence.

**Fig. 4:**
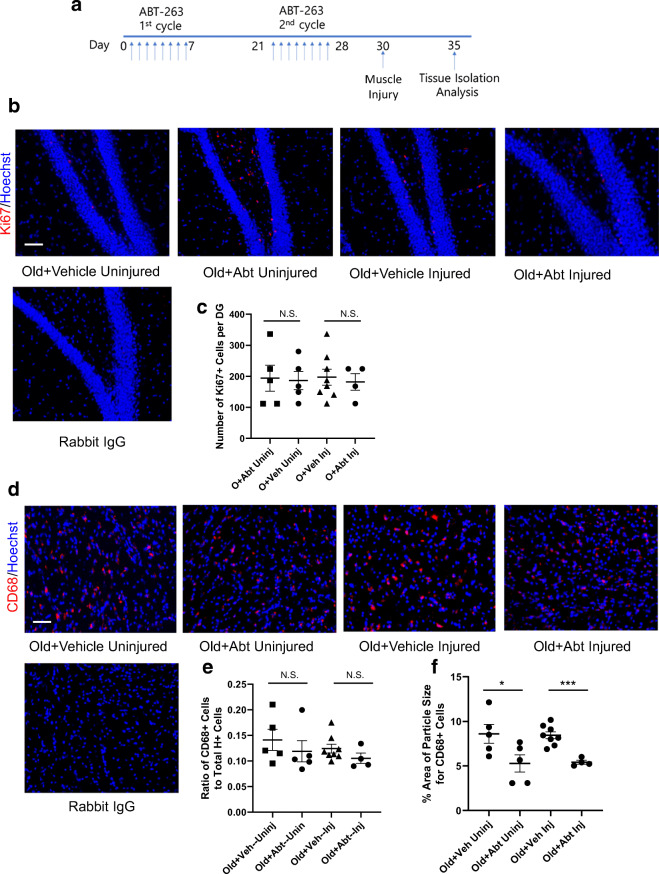
Effects of ABT 263 senolytic on hippocampal neurogenesis and neuroinflammation of old mice. **a** Schematic of the study. There were two 7-day periods where mice were given ABT 263 or vehicle by gavage once per day. A 2-week interval followed between each 7-day gavage period. TA muscles of some mice were injected with cardiotoxin for experimental injury, while other animals were not injured. **b** Brains were snap frozen and serially cryosectioned at 25 μm. These sections were immunoassayed with anti-Ki67 antibodies (proliferation marker), while using Hoechst to counterstain all the nuclei. Representative images of the hippocampal dentate gyrus show Ki67 (red)/Hoechst (blue) double-positive cells in subgranular zone (proliferating SGZ NPCs). **c** Quantification of Ki-67^+^/Hoechst^+^ SGZ cells in the dentate gyrus was performed for both injured and uninjured cohorts. ABT 263 did not improve hippocampal neurogenesis in either cohort. Old + Veh Uninj versus O + Abt Uninj *p* value = 0.89; Old + Veh Inj versus O + Abt Inj *p* value = 0.72. *p* values were obtained by two-tailed Student’s *t* test. *N* of Old + Veh Uninj = 5, *N* of Old + Abt Uninj = 5, *N* of Old + Veh Inj = 8, *N* of Old + Abt Inj = 4. **d** Immunofluorescence was performed for CD68 (activated microglia marker). Representative images of CD68 (red)/Hoechst (blue) double-positive cells. **e** Quantification of CD68^+^/Hoechst^+^ cell frequency in the brain was performed for the injured and uninjured cohorts. ABT 263 did attenuate CD68^+^ cell frequency. Old + Veh Uninj versus O + Abt Uninj *p* value = 0.47; Old + Veh Inj versus O + Abt Inj *p* value = 0.21. N.S. non-significant. **f** CD68 cluster size per cell analysis was performed on Fiji. The size of CD68 clusters was significantly reduced by ABT 263. Old + Veh Uninj versus O + Abt Uninj *p* value = 0.05; Old + Veh Inj versus O + Abt Inj *p* value < 0.0005. *p* values were obtained by two-tailed Student’s *t* test. For all experiments: *N* of Old + Veh Uninj = 5, *N* of Old + Abt Uninj = 5, *N* of Old + Veh Inj = 8, *N* of Old + Abt Inj = 4. Scale bar = 50 μm. There was no non-specific fluorescence in isotype-matched IgG negative controls

Previous studies have shown that muscle injury by cardiotoxin (CTX) injections worsens hippocampal neurogenesis in mice [21, 29]. To interrogate these effects in the context of ABT 263 administration, some mice received CTX injury on day 30 of the treatment, while others have not (Fig. 4a). Neurogenesis and neuroinflammation were examined in the brains of these animals as published [18, 21, 29] and shown in Fig. 2.

Neurogenesis occurs in the subgranular zone (SGZ) of the DG declines with age and is shown to be influenced by systemic factors [5, 9, 10, 18, 21, 29]. Proliferation of neural precursor cells (NPCs) that are born in the SGZ of the DG was assayed by immunofluorescence of the proliferation marker Ki67 in serial brain sections (Fig. 4b). Neurogenesis was quantified throughout the thickness of each hippocampus (Fig. 4c). Consistent with the poor hippocampal neurogenesis that is typical old mice, the animals in the control group had ~ 150–200 NPCs per DG [18, 21, 29]. Interestingly, the number of proliferating NPCs in hippocampi of the aged mice which were treated with ABT 263 was not significantly different from that of the vehicle controls. The numbers of proliferating NPCs in the SGZ of ABT 263– or vehicle control–treated old mice were similar to those of OO cohort, and all were significantly lower than those of the ONBE group (Supplementary Figure 2A).

The extent of neuroinflammation was evaluated using CD68 as a marker for activated microglia (as in Fig. 2a). The relative frequency of CD68^+^ cells in ABT 263–treated aged mice remained as high as in the control group (Fig. 4d, e). The frequency of CD68^+^ cells was similar between ABT 263 and control vehicle cohorts and the OO cohort, and all were significantly higher when compared to CD68^+^ cell frequency in the ONBE cohort (Supplementary Figure 2B).

When performing these studies, we noticed that the magnitude of CD68 fluorescence appeared to be lower in ABT 263 cohort than in the control vehicle group (even though the numbers of the CD68^+^ cells did not change). Data quantification of CD68 clusters’ size per cell confirmed this observation and demonstrated a significant decline in the size of CD68 clusters upon the treatment of old mice with ABT 263 (Fig. 4f).

These results demonstrate that as compared to NBE, senolytic ABT 263 does not enhance hippocampal neurogenesis and has a weaker, but measurable effect on attenuation of neuroinflammation. These differences manifest even though both ABT 263 and NBE reduce brain senescence and systemically attenuate SASP.

### Evolutionarily conserved effects of NBE and TPE on systemic proteins with direct influence on the brain

Considering the uncovered multiple positive effects of NBE on brain health and function and the postulated rejuvenative effects of TPE, we performed comparative proteomics analyses on blood serum (mouse and human) that were isolated before versus after these procedures (6 days after NBE and 1 month after TPE). As mentioned above, the BBB becomes permeable to a number of systemic proteins with age (including TGF-beta ligands and albumin [45–47]), suggesting an increased capacity of peripheral proteins to act in the old brain.

Starting with proteomics on systemic factors that were modulated by NBE or TPE and showed > 2-fold change with *p* < 0.05, we focused on the comparative levels of the proteins that directly regulate brain health, including receptors that are present on neurons, microglia, or other brain resident cells; ligands that influence proliferation and/or differentiation of neural cells; and signaling proteins that affect neuroplasticity or are neuroprotective.

**Fig. 5:**
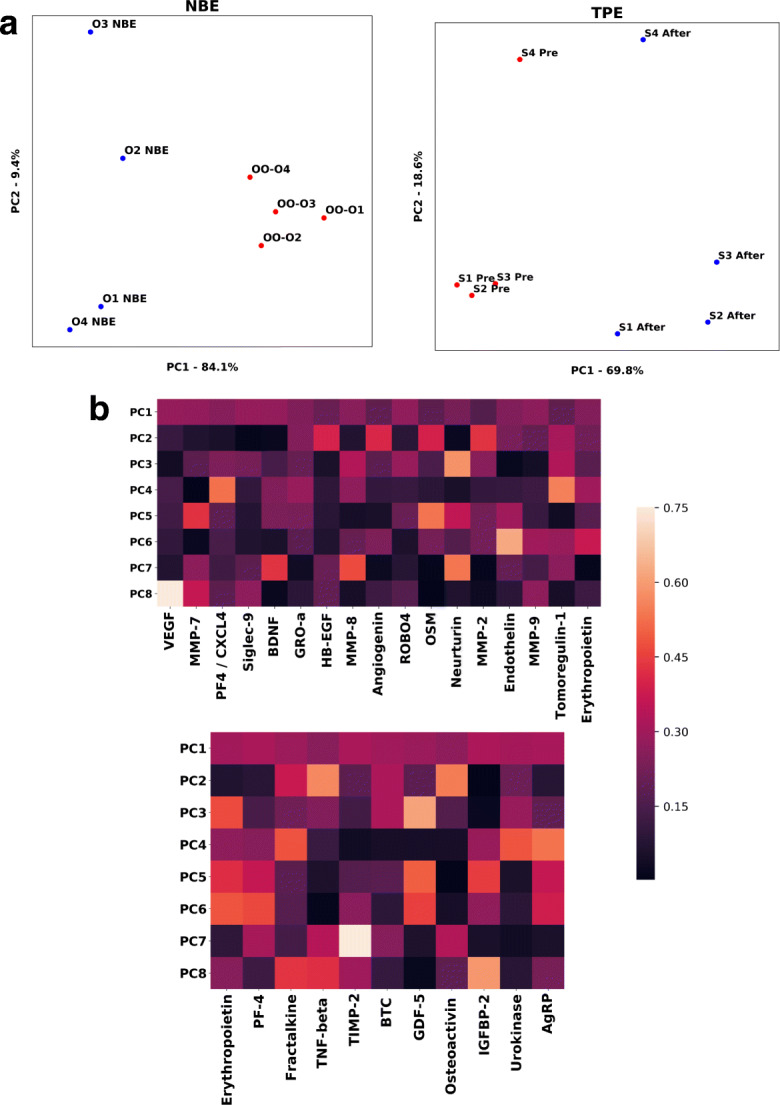
Multiple determinants of brain health and function become simultaneously elevated after NBE and TPE. **a** PCA was performed on an aggregate dataset of serum proteomics of control old mice (old exchanged with old) and old animals that underwent one round of NBE (serum was collected 6 days after NBE), with each point representing a unique mouse whose value is computed by a PCA transformation, using as features eleven proteins that play a direct effect in brain health. In this PCA visualization, the aggregation of OO proteome is separated from the aggregation of ONBE proteome. The first two principle components account for 93.5% of the variation between cohorts. **B.** PCA was performed on human serum proteomics, comparing Pre TPE samples with those collected at 1 month after a single procedure, using as features 17 brain health determinants. The first two principle components account for 88.4% of the variation before and after treatment. **C.** Heat maps for the loading scores for each principle component for the 17 determinants in human (top) and the 11 determinants in mice (bottom). Erythropoietin and PF4 were evolutionarily conserved proteins influenced by NBE and TPE. All serum samples were the same as in [18].

These studies identified 15 proteins that are directly significant for brain health, maintenance, and repair and that are changed in the TPE proteome, and 11 such proteins changed in the mouse NBE proteome. Interestingly in both species, dilution of old plasma ultimately resulted in a lasting elevation (not decline) of these determinants, which is consistent with our general conclusion on a resetting of the molecular signaling milieu to health (Fig. 5).

Interestingly, proteins that influence multiple parameters of brain health were simultaneously modulated by the NBE and TPE, therefore providing a mechanism for improving multiple brain health aspects at once: enhancing brain function and synaptic plasticity, improving neuroprotection, attenuating neuroinflammation, diminishing neurotoxicity, and enhancing proliferation and differentiation of neural stem cells (Table 1).

**Table 1:** Proteins simultaneously modulated by the NBE and TPE

	Effects on the brain
TPE elevated	
BDNF	Neurogenesis, neuroprotection, plasticity related to learning and memory [53]
Tomoregulin1	Neuroprotection: binds amyloid-β (Aβ) [54]
VEGF	Neuroprotection, secreted by neurons [55]
MMP-2, 7, 8, 9	Synaptic re-organization and memory [56]
Siglec	Neuroprotection; alleviates microglial toxicity [57, 58]
GRO-a	Oligodendrocyte proliferation and migration [59]
HB-EGF	Neuroprotection and function [60]
Angiogenin	Neuroprotection [61]
Robo4	Neurogenesis [62]
OSM	Neuroprotection and homeostasis of neural precursor cells [63]
Neurturin	Neuroprotection and neurogenesis [64]
Endothelin	Functional activity: neurotransmission [65]
NBE elevated	
Platelet factor 4	Neurogenesis [66]
Erythropoietin	Neuroprotection [67]
Fractalkine	Homeostasis: neuron/glia crosstalk [68]
TNF-β	Neuronal plasticity, synaptic scaling, hippocampal neurogenesis [69]
TIMP-2	Inhibits neuronal proliferation and promotes the differentiation [70]
Betacellulin	Neurogenesis [71, 72]
GDF5	Neurogenesis and neuroprotection [73, 74].
Osteoactivin	Neuroprotection: expressed on microglia—phagocytosis of myelin debris [75]
IGF-BP2	Neuronal plasticity, learning, and memory as well as information processing [76, 77]
uPA	Neuroprotection and repair [78, 79]
AgRP	Functional activity [80, 81]

These results confirm and extrapolate the positive effects of old plasma dilution on the brain by demonstrating a lasting recalibration of systemically present molecular determinants that have direct effects on brain health and function.

## Discussion

Plasma exchange is currently FDA approved for other diseases, and based on our recently published and current work, TPE yields great therapeutic potential toward treating age-associated degenerative and inflammatory diseases of the brain, as well as general age-imposed decline in brain health and function.

The use of TPE, where the patient’s plasma is filtered and replaced with a 5–20% albumin solution, was investigated for its effectiveness to mitigate the progression of mild-to-moderate Alzheimer’s disease. A phase I study that began in 2005 and concluded in 2009 revealed that cognition scores and cerebrospinal fluid Aβ changed little, yet hippocampal volume and frontal and temporal cortex perfusion increased in a 6-month follow-up [82, 83].

A phase II study beginning in 2007 and concluding in 2017 consisted of a larger patient population, measured the same parameters as phase I, and involved a more intensive plasma exchange regimen. Results from phase II were remarkable in attenuation of cognitive decline, with either close to or at 95% confidence interval. Additionally, stabilization of perfusion in the frontal, temporal, and parietal areas was observed in plasma exchange patients [83–85]. However, behavioral, functional, and cognitive improvements were not determined with statistical significance [86]. Current multicenter trials are being conducted to further investigate the efficacy of TPE in treating Alzheimer’s disease [83, 86].

Our results agree with the promising direction of TPE being applied to treating Alzheimer’s disease; moreover, our work expands the rejuvenative phenotypes, suggesting that brain diseases, as a class, and even physiological brain aging can be at some point prevented, attenuated, and restored to health–youth through an appropriate dilution of old blood plasma or factors.

Furthermore, we outline systemic candidate proteins that might be responsible for the positive effects of TPE/NBE on the brain, thereby suggesting future therapeutic avenues for agonist/antagonist approaches to improve brain health and reduce brain aging.

In this regard, previous studies have shown that attenuation of TGF-beta signaling by Alk5 inhibitor, particularly, when combined with ectopic oxytocin, improves hippocampal neurogenesis, diminishes neuroinflammation. and improves cognition in aged mice [13, 29]. Effects of NBE are overall stronger than those of Alk5 inhibitor plus oxytocin, which is expected, as old plasma dilution acts by multiple mechanisms and elevates the levels of numerous “youthful” systemic factors [18].

Comparing NBE with another prominent pharmacological rejuvenation modality, senolytics (ABT 263 (Navitoclax) which induce apoptosis in senescent cells [87]) revealed that plasma dilution has much stronger positive effects on the old brain than ablation of senescent cells.

There is substantial interest in developing senolytic therapies to treat diseases that are associated with age. ABT 263 is of great clinical interest since it was initially used to treat various forms of cancer [88–91] and has effect on most peripheral tissues, but must be primed with additional drugs in order to affect brain tumors [92, 93].

The fact that NBE has a stronger effect than ABT 263 in brain rejuvenation suggests that resetting signaling milieu to health–youth by old plasma dilution is more robust for brain rejuvenation than attenuation of SASP through ablation of senescent cells. Interestingly, our data demonstrates that brain senescence is diminished by both peripherally acting ABT 263 and NBE, in support of the notion that age-elevated systemic senescence and SASP might induce central senescence [45, 46, 51, 52]. The CD68 cluster size reduction, observed in this work, might be associated with a modest attenuation of neuroinflammation [94] by ABT 263. And, one of the proposed mechanisms for the amelioration of Alzheimer’s disease indeed involves a reduction in neuroinflammation where peripheral modulation impacts the central brain inflammation [83]. It would be interesting to examine if ABT 263 might be effective in such applications.

Recent studies show that experimental brain injuries in mice negatively affect hippocampal health (loss of synapses, neuronal death) and impact cognition even when the foci of such injuries and subsequent microglia activation are elsewhere in the brain [95–98]. Moreover, local microglia activation leads to broad inflammatory responses in the brain through autocrine and paracrine functions of the pro-inflammatory secretome, which cumulatively increases neuroinflammation throughout the brain [99, 100]. In concert with our findings, an injury sustained in the thalamus (e.g., one of the regions where we found an age-associated, NBE reduced neuroinflammation) activates microglia and perpetuates neurodegeneration and cognitive decline [101]. Together, these findings suggest that microglia activation is diffuse, spreading from one brain region to others.

Our study has mapped age-elevated frequencies of CD68^+^ cells to the thalamus, the hypothalamus, and the midbrain. Age-associated microglia activation in each of these regions is indeed causal in brain pathologies and is expected to affect brain function and cognitive performance [102–105]. And in general, neuroinflammation is, at least in part, responsible for the age-related loss of neurons [106].

Therefore, the rapid cognitive improvements of old mice in this study are thought to arise from abrogating (through NBE) the otherwise age-increased extent of neuroinflammation. Of note, plasma dilution might be also clinically relevant to the attenuation of hyper-activated CD68^+^ microglia in psychiatric diseases, where neuroinflammation is implicated in pathology [107].

It is highly unlikely that increased neurogenesis in aged mice by NBE [18] contributes to the observed here improvement in cognition. As was the case previously [29], we assayed cognitive performance 1 week after a single procedure of NBE; thus, there was not enough time for the new neurons to be formed and/or integrate into the networks.

Summarily, this work supports the paradigm that diluting and resetting to health and youth systemic signaling milieu promotes the “young” determinants of tissue health, rejuvenating the brain (and in fact, all tissues and parameters that were studied up-to-date [18]).

## Materials and methods

### Animals

All in vivo experiments and procedures were performed in accordance with the policies set by the Office of Laboratory Animal Care and under the approved protocols at the University of California, Berkeley, and the Buck Institute for Research on Aging. Young male C57BL/6 mice (2 months old) were purchased from Jackson Laboratory while aged mice (18 months old) were purchased from the National Institute of Aging (NIA). The aged mice were allowed to acclimate at the same animal facility that housed young mice for several weeks prior to the studies. All mice were fed identical diets. All aged mice used in the OO, ONBE, Old + vehicle, and Old + ABT 263 cohorts were obtained from the NIA.

### Number of animals (*N*)

A power analysis was performed in order to determine the sample sizes for the experiments presented as described previously [18].

### Jugular vein cannulation surgery and blood exchange procedures

Note that all equipment used for these procedures were autoclave sterilized. A bead sterilizer was used upon repeated contact with multiple mice. Jugular vein cannulation and blood apheresis procedures were performed as previously published [18, 21]. Briefly, mice were given buprenorphine (0.1 mg/kg) and anesthetized with 1–3% isoflurane in oxygen to full relaxation. Ophthalmic ointment was applied to each eye to prevent drying. Mice were shaven around their necks, rested in dorsal recumbency, and betadine surgical scrub was applied to their bare skin three times. An isopropanol wipe was used to remove the betadine after each betadine application. The mice were then placed on a sterile field. Once there was no reaction to toe pinch, a 1–1.5-cm incision was made to the right of the midline and the right internal jugular vein was isolated. A 6-0 silk suture was used to ligate the cranial end of the vein. Gentle tension was applied to the ligated end of the vein, while another 6-0 suture was passed underneath the vein and loosely knotted. A 25-gauge needle with its beveled end bent outward to 90° was used to perform the venotomy. The 1-Fr end of a pre-heparinized 1–3-Fr catheter (Instech Labs, C10PU-MJV1403) was promptly inserted into the jugular vein and the caudal ligature was tightened to hold the catheter in place. Once patency was confirmed, the catheter was plugged, and an additional cranial ligature is made to latch the catheter in place. Mice were then rested in left lateral decubitus to thread the catheter between their scapulae. To accomplish this, blunt forceps were used to create space underneath the skin, passing the incision site to the scapulae. A 16-guage needle was positioned between the scapulae and inserted underneath the skin at the level of the incision site. The 3-Fr end of the catheter was passed through the 16-guage needle. Reflex 7 wound clips were used to close the incision site, and the catheter protruding the skin was secured with a drop of Dermabond. The mice were taken off anesthesia, given meloxicam subcutaneously (5 mg/kg) for 7 days post-procedure, and allowed to recover in their caged. Antibiotic ointment with lidocaine was applied to the closed site. The mice were taken off anesthesia and dosed with subcutaneous meloxicam (5 mg/kg s.q.) for 7 days post-procedure.

Blood from young or aged donor mice was obtained by a terminal cardiac puncture and anti-coagulated with 3 units of heparin. Blood samples were centrifuged spun at 500*g* for 5 min. The platelet-rich plasma fractions were carefully removed; blood cell pellets were resuspended in normal saline and then spun down once more at 500*g* for an additional 5 min. The saline layer was removed, and blood cell pellets were then resuspended in an equal volume of 5% MSA in normal saline, 0.9% sodium chloride. The replacement albumin is > 95% pure by manufacturer’s analysis (https://mol-innov.com/products/albumin-mouse-plasma/). These blood mixtures were passed through a 50-μm FACS mesh cap tube in order to de-clump cells and filter out any clots. Extracorporeal blood exchanges were performed between pairs of young mice, pairs of old mice, and young mice or old mice and a tube containing synchronic blood cells in 5% MSA several hours following the surgeries. These designer blood solutions were prepared immediately prior to performing blood exchanges.

Blood exchanges were performed 24 h after cannulation surgeries to allow for adequate recovery. The mice were anesthetized once more with 1–3% isoflurane in oxygen and given ophthalmic ointment while resting in ventral recumbency. Catheters were assessed for patency and flushed with 3 units of heparin saline. A total of 150 μL of blood was exchanged between the mouse and a tube of our designer blood solution 15 times for the 50% replacement of blood plasma with saline + albumin fluid, or for 50% synchronic young or old blood exchanges, as in [21]. The exchange process lasts approximately 30–40 min. Once completed, the catheters were plugged, the mice were taken off anesthesia, and they were allowed to recover.

### ABT-263 treatment

For drug treatments, 22- to 24-month-old male C57BL/6 mice from the National Institute on Aging (Bethesda, MD, USA) were treated with vehicle or ABT 263 (APExBIO, USA) diluted in 10% ethanol, 30% polyethylene glycol 400, and 60% Phosal 50 PG (Lipoid, Germany). ABT 263 was administered by oral gavage at 50 mg per kg body weight per day (mg/kg/day) for 7 days per cycle for two cycles with a 2-week interval between the cycles.

### Cardiotoxin muscle injury

Mice were injured by intramuscular injections of CTX (Sigma, 10 mL per muscle at 0.1 mg/mL) into the tibialis anterior (TA) and gastrocnemius (GA), as previously published [1, 18, 21, 29].

### Tissue isolation

Mice were sacrificed per the guidelines of UC Berkeley’s and Buck Institute’s OLAC administration. Blood was collected by terminal cardiac puncture and was allowed to clot completely at room temperature for at least 30 min. Clotted blood samples were centrifuged at a speed of 5000*g* for 5 min in order to obtain serum. Post-mortem isolation of brain was performed. Tissues were embedded in Tissue-Tek optimal cutting temperature (OCT, Sakura Finetek, The Netherlands) and snap frozen in isopentane cooled to − 70 °C with dry ice.

### Tissue sectioning and brain mapping

OCT-embedded brains were sectioned with a cryostat. Coronal sections at 25 μm thickness were attached to gold-supplemented positively charged glass coverslip slides. The cryostat was used to locate the midbrain, thalamus, and hypothalamus regions as stated above. These regions of the brain were located approximately 4.5–4.8 mm from the most posterior edge of the cerebellum. Subsequent tissue sections were collected for another 1 mm passing this mark.

### Senescence-associated β-galactosidase staining

Senescence-associated β-galactosidase (SA-β-gal) staining was performed with a commercial Senescence β-Galactosidase Staining Kit (9860S, Cell Signaling Technology) according to the manufacturer’s protocol. Briefly, frozen old mouse brain sections were fixed in the fixative solution (provided in the kit) for 15 min at room temperature. After washing twice with PBS, the sections were stained with the β-galactosidase staining solution at 37 °C overnight in a dry incubator (Thermolyne, type I42300 Incubator, USA). After staining, the images were captured using an Axio Imager A1 microscope (Zeiss, Germany) at a magnification of × 200. The percentage of senescent area was determined by dividing blue-stained SA-β-gal-positive areas by the total area of each section, using the ImageJ software.

### Antibodies and labeling reagents

The following antibodies were used at 0.5–1 μg/mL:

- CD68: Abcam, Rabbit, ab125212, 1:500
- Ki67: Abcam, Rabbit, ab16667, 1:200
- Isotype-matched IgGs: Sigma-Aldrich, Rabbit, 1:1000
- Donkey anti-rabbit Alexa 546: Life Technologies, Invitrogen, Eugene, Oregon, A10040, lot #1946340, 1:2000
- Hoechst dye was used to stain DNA: Hoechst 33342, Sigma-Aldrich (B2261), 1:1000

### Immunofluorescence of brain samples

Mouse brain was serially sectioned into 25-μm-thick sections that were then fixed in 4% paraformaldehyde for 4 min at room temperature. Subsequently, these sections are then rinsed with 1× phosphate-buffered saline (PBS) several times (2–3 min per rinse) and then permeabilized with 0.1% Triton-X over ice for 5 min. Samples were then rinsed and blocked with PBS and 1% staining buffer 3 times (2–3 min per rinse). Brain samples were then treated with primary antibodies and left to incubate overnight at 4 °C. During the following day, sections are then washed 3 times (2–3 min per rinse) with staining buffer and then coated with secondary antibodies as described and incubated for 2 h. Samples were then washed 3 times with staining buffer (2–3 min per rinse); then, 2 droplets of Fluoromount (Sigma F4680) mounting media were added and coverslips were then placed on top of samples. All tissue sections for immunofluorescence studies were mounted on positively charged gold SuperFrost slides.

### Behavioral assays

The whisker-dependent texture discrimination test was performed as previously described [27, 28], with the encoding, resting, and testing phase lasting 10, 5, and 5 min, respectively. The novel object recognition (NOR) test was also conducted as previously described [27–29] with the encoding, resting, and the testing phases set to 10 min, 2 h, and 10 min, respectively. Such modifications of encoding and testing duration are intended to accommodate the slow movement of aged mice. Behavioral analyses were performed with the analyst blinded to the identity and the conditions (age, type of treatment) of the mice.

### Principle component analysis

Principle component analysis was performed via singular value decomposition with the Python-scikit-learn decomposition package. The data was scaled using StandardScaler in the pre-processing method sklearn, and the features were normalized by mean protein expression. Python-Matplotlib was used to visualize the PCA-transformed data, and Python-Seaborn was used to generate the heat maps used to visualize the PC loading for each protein feature.

### Data quantification and statistics

Neurogenesis was quantified by counting the number of Ki67^+^/H^+^ cells in 200 μm of the SGZ from each mouse from multiple as previously described [18, 29]. Neuroinflammation was scored by counting the number of CD68^+^ cells relative to the total number of nuclei counted per field of view. Mapping strategies have been described in Fig. 2. All analyses were performed on tissue sections that were imaged at × 20 magnification. Non-paired, one-tailed, and two-tailed (as appropriate) Student’s *t* tests were performed in GraphPad Prism 8 for all tissue analysis data.

## Supplementary information

ESM 1 (DOCX 4657 kb)

## Data Availability

All relevant data was provided upon the submission of this manuscript.
