# Supplementary material for: Plasma dilution improves cognition and attenuates neuroinflammation in old mice
Source: GeroScience. 2020 Nov 15;43(1):1–18. doi: 10.1007/s11357-020-00297-8 (PMC8050203; doi:10.1007/s11357-020-00297-8)
Supplement: Supplementary file 1 — (DOCX 4657 kb) [file 11357_2020_297_MOESM1_ESM.docx]

**Supplementary Figures**


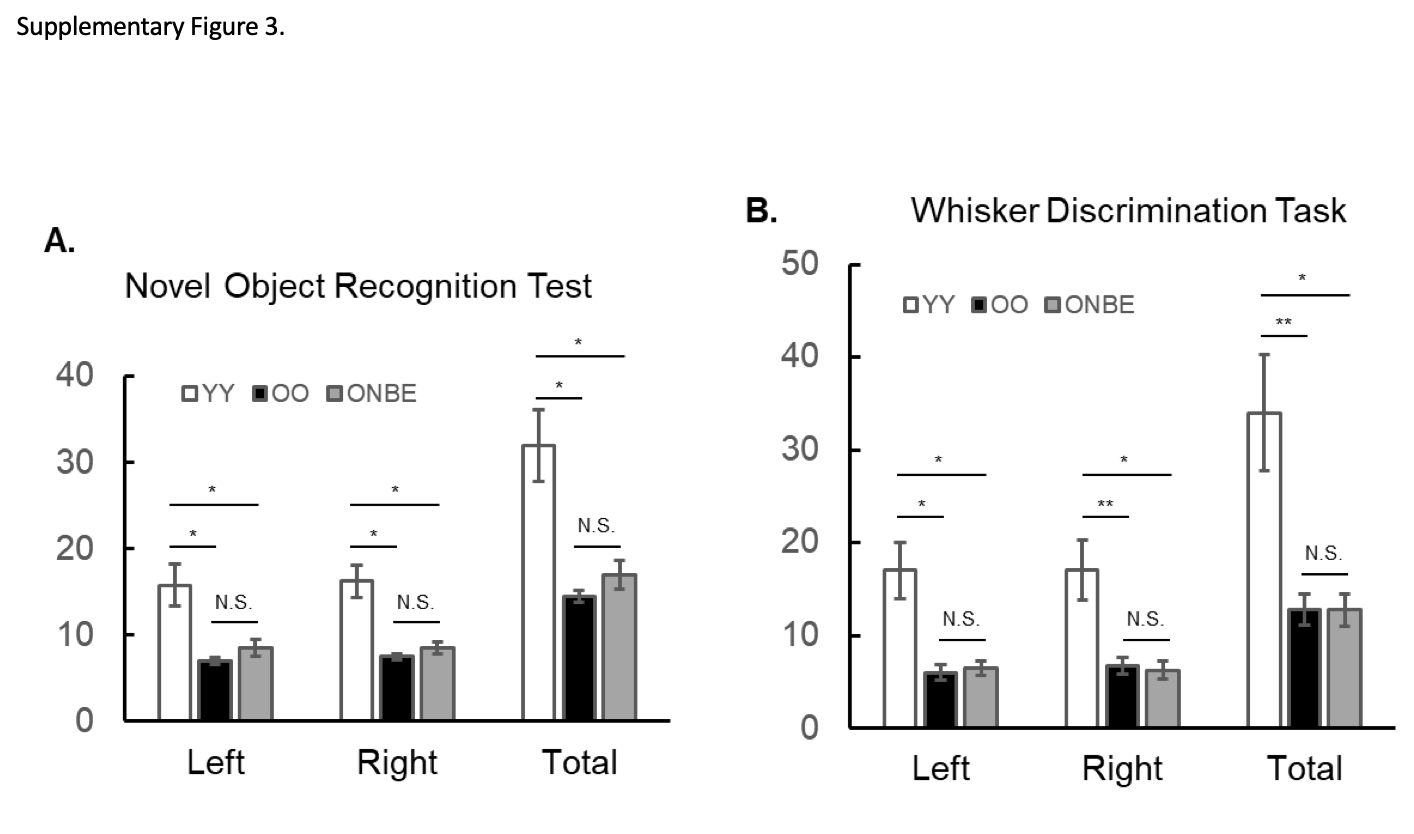


**Supplementary Figure 1. Encoding phases of mice from each cohort for the behavioral assays**

**A.** Counts for the number of approaches that each animal has made to the left or right object during the encoding phase for novel object recognition. As expected, YY mice have approached each and both objects more often than either OO old ONBE animals. Left p-values: YY vs OO *p-value = 0.0167, YY vs ONBE *p-value = 0.01854, OO vs ONBE N.S. p-value = 0.7958. Right p-values: YY vs OO *p-value = 0.02762, YY vs ONBE *p-value = 0.02542, OO vs ONBE N.S. p-value = 0.8269. Total p-values: YY vs OO *p-value = 0.02089, YY vs ONBE *p-value = 0.02148, OO vs ONBE N.S. p-value = 1. **B.** Numbers of approaches were obtained as in A but for the whisker discrimination task. As in A, YY mice have approached the textured objects more often than animals of both older cohorts. Left p-values: YY vs OO *p-value = 0.01347, YY vs ONBE *p-value = 0.04562, OO vs ONBE N.S. p-value = 0.41356. Right p-values: YY vs OO *p-value = 0.00376, YY vs ONBE *p-value = 0.01233, OO vs ONBE N.S. p-value = 0.46799. Total p-values: YY vs OO *p-value = 0.006654, YY vs ONBE *p-value = 0.02308, OO vs ONBE N.S. p-value = 0.4304.

**
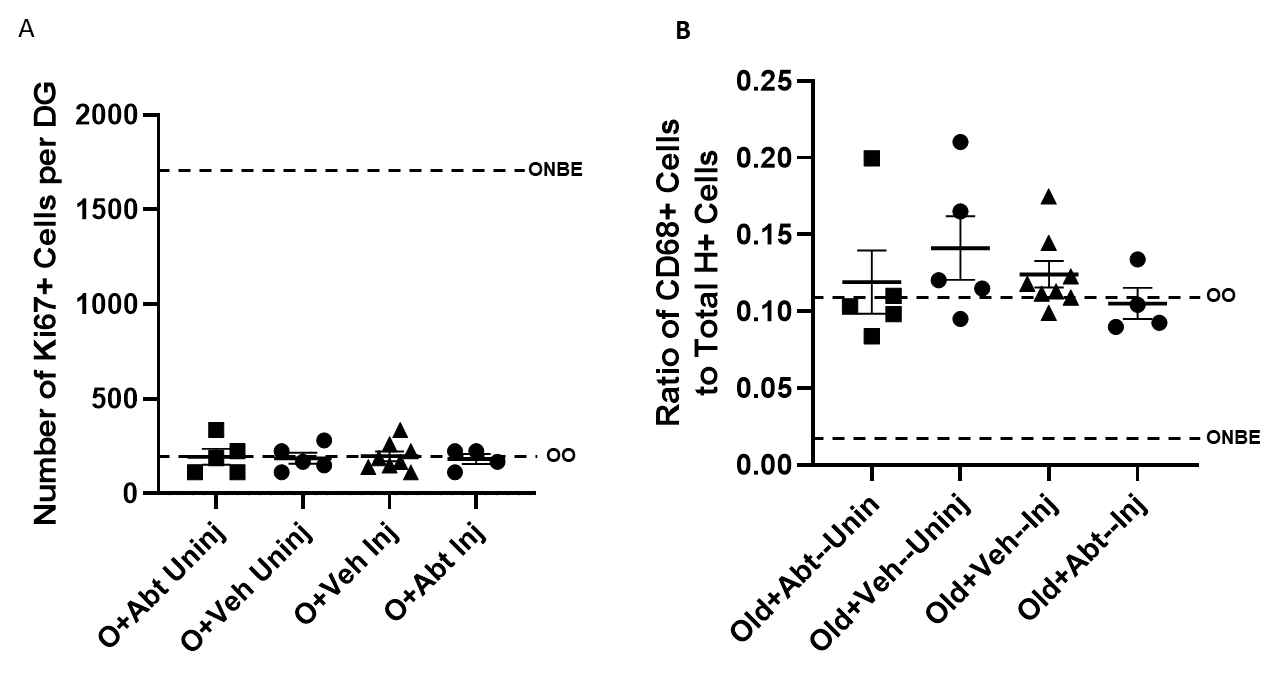
**

**Supplementary Figure 2. Comparison of NBE vs ABT 263’s effects on hippocampal neurogenesis and neuroinflammation.**

Dashed lines indicate means of ONBE and OO cohorts for each panel, respectively. **A.** Hippocampal neurogenesis and **B.** neuroinflammation are substantially improved by NBE. These parameters for aged mice treated with ABT 263 or vehicle remain similar to those of the OO cohort indicating that ABT 263 had no rejuvenative effects in the studied features.
